# Role of platelet rich plasma in management of early knee osteoarthritis pain: A retrospective observational study

**DOI:** 10.1016/j.inpm.2023.100297

**Published:** 2023-11-28

**Authors:** Sandeep Khuba, Dheeraj Khetan, Sanjay Kumar, Keshav Kumar Garg, Sujeet Gautam, Prabhaker Mishra

**Affiliations:** a Department of Anaesthesiology, Sanjay Gandhi Postgraduate Institute of Medical Sciences, Lucknow, India; b Department of Transfusion Medicine, Sanjay Gandhi Postgraduate Institute of Medical Sciences, Lucknow, India; c Department of Anaesthesiology, Healthworld Hospital, Durgapur, India; d Department of Biostatistics and Health Informatics, Sanjay Gandhi Postgraduate Institute of Medical Sciences, Lucknow, India

## Abstract

**Introduction:**

Knee joint osteoarthritis is a well-known cause of pain and disability in patients above 40 years of age. It is treated by use of non-steroidal inflammatory drugs, corticosteroids, glucosamine, chondroitin sulfate, physiotherapy with limited success. The platelet rich plasma (PRP) contains a large amount of platelet derived growth factors, cytokines and anti-inflammatory molecules which showed promising results in recent studies to relieve pain of knee joint osteoarthritis. The present study aims to determine the efficacy of intraarticular PRP for pain relief and functional improvement in patients with early knee joint osteoarthritis.

**Methods:**

It is a retrospective observation study involving patients who underwent single intraarticular administration of PRP for knee pain with Kellgren-Lawrence (KL) grades I or II knee joint osteoarthritis. The Visual analogue scale (VAS) score and Oxford knee score (OKS) were recorded pre-procedure and at 1- and 6-month post-procedure.

**Results:**

A total of 31 patients (20 females, 11 males) underwent PRP therapy for knee pain (16 kL grade I, 15 kL grade II). The mean age and duration of symptoms were of 53.9 years (range: 79–42 years) and 5.53 ± 2.35 years respectively. There was a significant reduction (p < 0.05) in VAS scores from pre-procedure (68.06 ± 8.33) to post procedure at 1 month (37.74 ± 11.16) and 6 months (54.52 ± 11.78). There was also significant improvement (p < 0.05) in OKS score from pre-procedure (31.1 ± 3.47) to post-procedure at 1 month (39.06 ± 3.37) and 6 months (34.10 ± 3.75). No adverse effects were reported in patients during the study period.

**Conclusion:**

This small retrospective study suggests that a single administration of intraarticular PRP may be safe and effective for pain relief and functional improvement for up to 6 months in patients of early-stage osteoarthritis.

## 1 Introduction

Osteoarthritis (OA) is a chronic multifactorial degenerative condition defined by loss of articular cartilage, marginal bone hypertrophy (osteophytes) and a number of biochemical and morphological changes to the synovial membrane and joint capsule [1]. OA is the most common form of arthritis and often affects the hands, knees, feet, and hips. Knee OA is a well-known cause of disability around the globe affecting both physical and mental health of patients. Prevalence of radiographically confirmed symptomatic knee OA has been estimated at 3.8% globally, with rates being higher in women (4.8%) compared with men (2.8%) [2]. Knee OA accounts for approximately 80% of the disease's total burden [3]. The overall prevalence of knee OA is 28.7 % in Indian population [4].

Recent research has identified several key biochemical pathways that could be targeted therapeutically through biological interventions [5]. Treatment options include both invasive and non-invasive modalities. Drug therapies, including analgesics, corticosteroids, glucosamine, chondroitin sulfate, and non-steroid anti-inflammatory drugs are used to relieve pain and symptoms as well as slow the progression of arthritis [6]. Intraarticular injections like Hyaluronic acid [7] and Platelet rich plasma [8] have been recently studied for effectiveness in knee osteoarthritis.

Platelet rich plasma (PRP) is an autologous concentration of human platelets in a small volume of plasma, having regenerative effect and has been shown to provide some symptomatic relief in early OA of the knee [9]. NICE guidelines confirm that PRP injections, being a minimally invasive intervention for knee osteoarthritis, raise no major safety concerns, but the evidence on its effectiveness is inadequate and requires further research [10].

Autologous platelet-rich preparations containing a large pool of growth factors (GFs) and proteins like platelet-derived growth factor, transforming growth factor beta, vascular endothelial growth factors, endostatins, platelet factor 4, angiopoietins, and thrombospondin 1 are mainly stored in the alpha granules. These are secreted upon activation of platelets and have been implicated in the healing process [11]. Platelets have also been identified to have analgesic properties by releasing protease-activated receptor 4 peptides [12].

## 2 Aims and objectives

The present study intends to evaluate the effectiveness of single dose of leukocyte rich platelet rich plasma in early knee osteoarthritis pain in terms of pain relief and improvement in function.

## 3 Materials and methods

### 3.1 Study design and population

This retrospective observational cohort study is being conducted after approval from institutional ethical committee with IEC code 2020-269-others-EXP-30.

Setting: Pain clinic, Department of Anaesthesiology.

Study population: The Patients who underwent PRP injection for early knee osteoarthritis pain from year 2015–2019.

Inclusion criteria: The patients who underwent single intra-articular PRP administration in the knee joint for early knee osteoarthritis pain. The early knee osteoarthritis included knee osteoarthritis of Kellgren & Lawrence Grade I–II. The Kellgren-Lawrence (KL) scale, has been used since 1957 to assess the severity of radiographic knee OA based on several radiographic features of OA, including joint space narrowing and osteophyte development [13].

Exclusion criteria: The patients who underwent PRP therapy with a history of systemic autoimmune rheumatic diseases, use of corticosteroids within 3 months of PRP injection, and knee osteoarthritis with Kellgren & Lawrence Grade III–IV were excluded.

Primary outcome of interest: Visual analogue scale (VAS) scores for pain and Oxford knee score (OKS) score.

Secondary outcome of interest: Any adverse or side effects following PRP therapy.

Assessment: VAS score for pain is calculated by a 0–100 mm horizontal line where 0 corresponds to no pain and 100 to severe pain. The patient is asked to mark his or her current pain on this line. The point where the patient has marked this line is measured and reported as the score. Oxford knee score is a self-administered questionnaire that consists of 12 questions relating to the pain and functional status of the patient. Each question is scored from 1 to 5 with the severity of response increasing with the score. The minimum score is 12 and the maximum score is 60. The lower is the score, the better the pain relief for the patient. The assessment was done pre-procedure and post-procedure at 1 and 6 months.

Source of data: The hospital records of these patients were reviewed for demographic and clinical data.

### 3.2 Procedure

PRP preparation: Leukocyte richPRP (L-PRP) [14] was prepared from autologous whole blood donation. 150 ml of whole blood was collected from individual patients into a citrate anticoagulant containing single blood bag (Terumo Penpol blood bag, 150 ml). A 100 ml transfer bag (Terumo penpol blood bag, 100 ml) was then attached to this primary bag using a sterile connecting device (Fresenius Kabi, Homburg, Compo Dock). This blood bag assembly was first subjected to a soft spin (890 rpm for 9 min at 22 °C) using refrigerated centrifuge (Thermo Fisher Scientific, Heraeus Cryofuge 6000i) to separate out layers of platelet rich plasma on top and red cells at the bottom. PRP was expressed out into the transfer bag using a manual expressor (Baxter USA, Fenwal 4R4414). The PRP bag was then subjected to a hard spin (3500 rpm for 5 min). This separated out PRP and platelet poor plasma (PPP). A small amount of PPP was added back to the primary bag leaving approximately 15–20 ml in the PRP bag. PRP was left undisturbed for 2 h to allow dissolution of all platelet aggregates. Then it was kept in the platelet agitator (Terumo Penpol PI400) overnight at 20–24 deg C. On day 1 of preparation, quality control testing (4–6 times increase in concentration of platelet count compared to baseline) was done using a three-part differential cell counter (Medonic- M series, Boule, Sweden).

Preparation of aliquots - PRP was divided into 3 aliquots of 5–6 ml each using sample diversion poches (Macopharma, France) with the help of a sterile connecting device. One to two aliquots were used for patient application while the remaining aliquot(s) was stored in the deep freezer ≤ - 40 °C for future applications.

PRP therapy**:** All the patients who underwent PRP therapy were asked to stop any NSAIDS use before 3 days of PRP therapy**.** On the day of procedure, the patient was positioned supine, with the knee in 90-degree flexion. The skin was disinfected with an antiseptic solution containing alcohol or iodine and draped. PRP was injected slowly under sterile conditions through the anterolateral “soft spot” with a 22-gauge needle in the knee joint. Gentle passive flexion and extension exercises of the knee were encouraged immediately after administration. To avoid a reduction in PRP efficacy, patients were advised not to take any nonsteroidal anti-inflammatory drugs (NSAIDs) or to apply ice locally for a week after injection.

Statistical analysis: Quantitative variables are presented in median (Q1, Q3) [Mean ± SD] {% change from the baseline} within parenthesis. Friedman Test (a non-parametric method for the repeated observations between three or more time points) was used to compare the OKS/VAS scores. The significant Friedman test (p < 0.05) was followed by Post hoc test (PHT) for the multiple comparisons using Bonferroni corrections. Multiple comparisons (All*) indicated that there was a significant change (p < 0.05) in scores from baseline to one month as well as from baseline to 6 months. Percent (%) changes (using mean score) of OKS and VAS are calculated at the time points of 1 month and 6 months respectively with respect to baseline. P < 0.05 was considered statistically significant.

## 4 Results

**Fig. 1:**
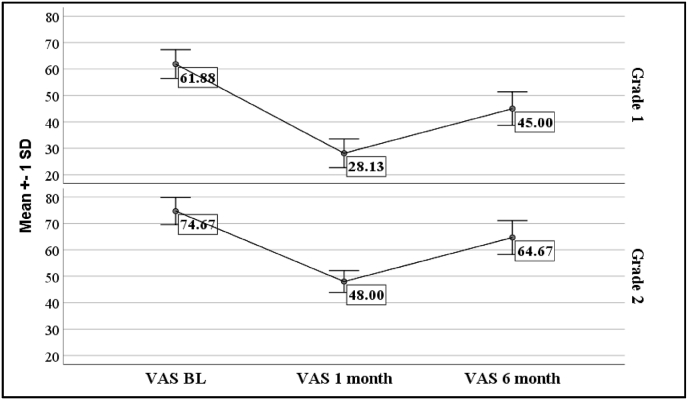
VAS scores at baseline and at 1 and 6 months.

**Fig. 2:**
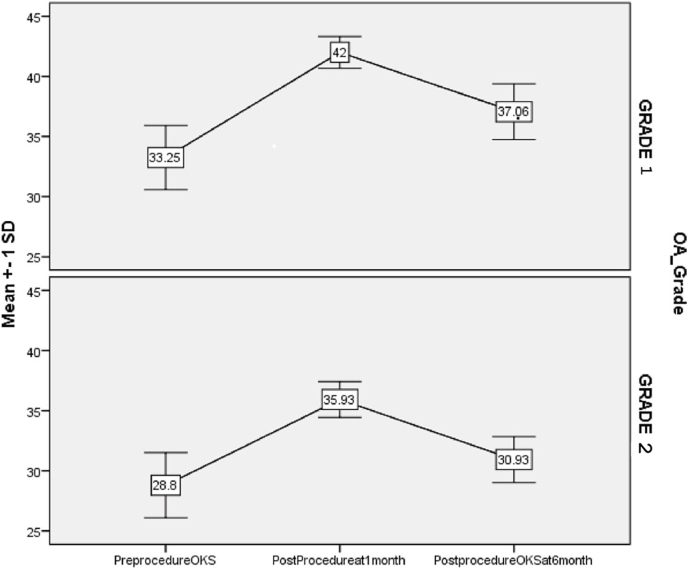
OKS at baseline and at 1 and 6 months.

A total of 31 patients with osteoarthritis were included in the study. Of the 31 patients, 20 (64.5 %) were females. The mean age and duration of symptoms were 53.9 years (range: 79–42 years) and 5.53 ± 2.35 years respectively. Plain radiographs taken prior to the procedure revealed grade I and grade II primary OA in 16 and 15 patients, respectively. The mean OKS score at baseline was 31.1 ± 3.47, while the mean VAS score before injection was 68.06 ± 8.33. There was significant improvement of VAS and OKS over baseline at 1 and 6 months as shown in Table 1. However, both OKS and VAS scores worsened from 1 to 6 months as shown in Fig. 1, Fig. 2. 48 % patients (95 % Confidence Interval: 30.15 %–66.94 %) achieved 50 % or more pain relief at 1 month post procedure. 42 % patients (95 % Confidence Interval: 24.55 %–60.92 %) achieved 50 % or more pain relief at 6 months post procedure. No adverse effects were noted during or after the PRP procedure.

**Table 1:** Distribution of OKS and VAS score during the patient follow up (N = 31).

Grades	N	Pre-procedure (Baseline)	1 month (1 M)	6 months (6 M)	P value	PHT* (p < 0.05)
**OKS**	31	31 (29, 34) [31.1 ± 3.47]	40 (36, 42) [39.06 ± 3.37] {25.59 %}	35 (30, 38) [34.10 ± 3.75] {9.65 %}	<0.001	All
Grade 1	16	33.5 (31, 35) [33.25 ± 2.67]	42 (41,43) [42 ± 1.32] {25.56 %}	37.50 (36, 38) [37.06 ± 2.32] {11.28 %}	<0.001	All
Grade 2	15	29 (27,31) [28.8 ± 2.7]	36 (35,37) [35.93 ± 1.49] {24.75 %}	30 (30,32) [30.93 ± 1.91] {7.40 %}	<0.001	All
**VAS**	31	70 (60, 70) [68.06 ± 8.33]	40 (30, 50) [37.74 ± 11.16] {-44.54 %}	50 (40, 70) [54.52 ± 11.78] {-20.36 %}	<0.001	All
Grade 1	16	60 (60,67) [61.88 ± 5.44]	30 (22.5,30) [28.13 ± 5.44] {54.54 %}	40 (40,50) [45 ± 6.32] {27.53 %}	<0.001	All
Grade 2	15	70 (70,80) [74.67 ± 5.62]	50 (50,50) [48 ± 4.14] {35.71 %}	70 (60,70) [64.67 ± 6.40] {13.39 %}	<0.001	All

Data presented in median (Q1, Q3) [Mean ± SD] {% change from the baseline} is given within parenthesis. Friedman Test used to compare the OKS/VAS score between three time points. The significant Friedman test was followed by Post hoc test (PHT) for the multiple comparisons. Multiple comparison (All*) showing the significant change in score from baseline to one moth as well as baseline to 6 months. Percent (%) changes (using mean score) are calculated at the time points of 1 month and 6 months respectively with respect to baseline **P < 0.05 significant.**

Mean platelet count, WBC count and red cell count of the PRP were 1164 ± 408 × 10^3^ cells/μl, 0.59 ± 0.27 × 10^3^ cells/μl and 0.06 ± 0.03 × 10^6^ cells/μl, respectively. No correlation (Pearson correlation) was observed between platelet or red cell count of PRP with the change in OKS or VAS scores at either one month or six month follow up (Table 2). WBC count of PRP however was found to have a negative correlation with the change in VAS scores at 6 month follow up (r = −0.58, p = 0.011, Pearson Correlation) as shown in Table 2. Similar negative correlation of WBC counts with change in VAS score at one month follow up was also observed; however it was not found to be significant (r = −0.446, p = 0.063, Pearson Correlation) (Table 2).

**Table 2:** Correlation between cell counts in PRP to that of patient's VAS and OKS scores at 1 and 6 months (N = 31).

		OKS 1 month	OKS 6 months	VAS 1 month	VAS 6 months
WBC count in PRP	Co-relation (r)	−.009	.153	−.446	−.586
	p-value	.971	.546	.063	.011
RBC count in PRP	Co-relation (r)	−.140	−.062	.09	.194
	p-value	.633	.833	.760	.507
Platelet count in PRP	Co-relation (r)	−.147	.13	−.05	−.154
	p-value	.429	.485	.979	.409

P-value <0.05 is significant.

## 5 Discussion

The present study shows that the use of a single dose of PRP lead to improvement in knee pain and function in patients suffering from early knee joint osteoarthritis for up to 6 months. We have shown that the use of PRP results in improvements in VAS and OKS scores for up to 6 months. Our results agree with the previous studies which have shown improvement in knee pain and function after PRP administration for up to 6 months [[15], [16], [17]].

Patho-physiologically in early osteoarthritis, matrix metalloproteases (MMPs), or degradative enzymes, are increased, which leads to an overall loss of proteoglycans and collagen. This is countered by tissue inhibitors of MMPs secreted by chondrocytes and increased synthesis of proteoglycans [18]. However, degradation is more, and this disequilibrium leads to a disorganized collagen pattern and loss of articular cartilage elasticity. There is a downregulation of anti-angiogenic cytokines, followed by upregulation of a cascade of pro-angiogenic cytokines [19]. Macroscopically these changes result in cracking and fissuring of the cartilage and ultimately erosion of the articular surface.

The growth factors present in PRP have been shown to promote cell recruitment, proliferation and angiogenesis resulting in a reduction in inflammation and expression of inflammatory enzymes [20]. PRP may induce a regenerative response by improving the metabolic milieu [21,22] and has also been observed to have a positive effect on chondrogenesis and mesenchymal stem cell proliferation [23]. In the present study we have used PRP product with platelet concentration more than 6 times normal, acting as a rich source of growth factors hence the observed pain relief.

The present study employed single administration of PRP to assess its effectiveness and safety. It has been observed that a single dose of PRP is as efficacious as 2 or 3 doses for pain relief for duration of up to 6 months [24]. After 6 months the beneficial effect of a single dose of PRP seems to lose at follow up of one year [25]. As far as the number of doses are concerned our study agrees with the previous studies as a single dose of PRP provides pain relief for up to 6 months [26].

Though, we have not used leukofilters or lymphocyte separation media during preparation of PRP, the WBC count in our PRP product was found to be lower compared to whole blood. There exists ambiguity regarding PRP terminology as no cut-off WBC levels have been mentioned in literature to consider PRP as LR or LP. Kobayashi et al. [27] used lymphocyte separation media for preparation of leukocyte poor PRP with a reported WBC count of 2.4 ± 1.3 × 10^3^/μL. Though the number of leukocytes in our study was very low it was still considered to be leukocyte rich in accordance with the classification given by Dohan et al. [14]. Some may, however, consider it to be leukocyte poor.

In our study we have used 6 ml of PRP concentrate with average platelet count of 1164 ± 408 × 10^6^/ml. Bansal et al. has demonstrated that single dose of at least 10 billion platelets in 8 ml volume leads to improvement in pain relief and function of knee joint for up to one year [26]. Due to the high platelet count in our PRP preparation, there was sustained pain relief for 6 months. Low platelet concentration PRP preparations have been mainly used in aesthetic and cosmetic surgery like for improvement of deep nasolabial folds [28,29]. However, some studies using low concentrate PRP for knee osteoarthritis have also reported sustained pain relief [30,31].

Because of the interaction between platelets and neutrophils, several in vitro studies have suggested that using leukocyte rich PRP could be effective in the treatment of knee OA. Platelets and neutrophils can prevent leukotrienes from being converted into lipoxin, triggering the resolution phase of the healing cascade [32,33]. In our study, we have used leukocyte rich platelet rich plasma. Similar studies and metanalysis have shown leukocyte rich PRP as effective as leukocyte poor PRP in knee osteoarthritis [34,35].

In our study we have used inactivated PRP as it has been observed that it increases proliferation of mesenchymal cells by 5-fold, improves cartilage and bone formation [36]. The use of activated PRP is associated with in-vivo inhibition of chondrogenesis and osteogenesis [37]. We have used PRP in patients with early stages of knee osteoarthritis as a greater efficacy of PRP has been observed in less severe or early grades of osteoarthritis [38,39]. Our results are in consistent with the findings of these previous studies. We have used only PRP for intraarticular administration as PRP has been shown to be more efficacious than hyaluronic acid, steroids or placebo [40]. In the present study, we have not observed any adverse effects in any of the patients.

## 6 Limitations of study

This is a retrospective observational study of small sample size with no comparison group. Many more patients underwent PRP injection during the study period with similar results. This study comprised 31 patients with grade 1 and 2 knee osteoarthritis who had follow-up data for up to 6 months. Patients with knee osteoarthritis grades 3 and 4 who underwent PRP were not included.

The duration of follow up was only for 6 months. So further studies with randomized controlled design are advised with follow up of more than 1 year duration to study the long-term efficacy and safety of PRP.

## 7 Conclusion

This small retrospective study suggests that a single administration of intraarticular PRP may be safe and effective for pain relief and functional improvement for up to 6 months in patients of early-stage osteoarthritis.

## Declaration of competing interests

The authors declare that they have no known competing financial interests or personal relationships that could have appeared to influence the work reported in this paper.
